# Collision-aware interactive simulation using graph neural networks

**DOI:** 10.1186/s42492-022-00113-4

**Published:** 2022-06-07

**Authors:** Xin Zhu, Yinling Qian, Qiong Wang, Ziliang Feng, Pheng-Ann Heng

**Affiliations:** 1 College of Computer Science, Sichuan University, Chengdu, 610065 China; 2 Shenzhen Institute of Advanced Technology, Chinese Academy of Sciences, Shenzhen, 518055 China; 3 Department of Computer Science and Engineering, The Chinese University of Hong Kong, Hong Kong, China

**Keywords:** Deep physical simulation, Collision-aware, Continuous collision detection, Graph neural network

## Abstract

Deep simulations have gained widespread attention owing to their excellent acceleration performances. However, these methods cannot provide effective collision detection and response strategies. We propose a deep interactive physical simulation framework that can effectively address tool-object collisions. The framework can predict the dynamic information by considering the collision state. In particular, the graph neural network is chosen as the base model, and a collision-aware recursive regression module is introduced to update the network parameters recursively using interpenetration distances calculated from the vertex-face and edge-edge tests. Additionally, a novel self-supervised collision term is introduced to provide a more compact collision response. This study extensively evaluates the proposed method and shows that it effectively reduces interpenetration artifacts while ensuring high simulation efficiency.

**Supplementary Information:**

The online version contains supplementary material available at 10.1186/s42492-022-00113-4.

## Introduction

Many computer graphics applications, such as computer games, movie production, and fashion design, require physical simulation. Traditional numerical calculation methods produce physically accurate and visually excellent results. However, these methods are time consuming. Consequently, they cannot satisfy the performance requirements for interactive applications.

Deep simulation methods have emerged as popular alternatives to traditional numerical calculation methods owing to the rapid development of deep learning techniques. These methods [1–4] use the ability of neural networks to learn nonlinear functions to propose differentiable models that output deformable objects as functions of the target shape, pose, motion, and other design parameters. However, these methods perform poorly in collision detection and response (CDR), which has a significant impact on visual realism and simulation accuracy. To avoid interpenetration, these methods manually set a relatively large collision threshold in the training data generation process [1]. However, a manually set threshold cannot meet the requirements for an accurate CDR.

This study proposes a framework for collision-aware interactive physical simulation using a graph neural network (GNN), which can achieve a CDR function similar to continuous collision detection (CCD), which is the most effective method for solving the CDR problem in traditional physical simulation. The GNN was used as the base model because it can provide complete vertex-edge-face information, which can be used intuitively in basic geometric primitive collision tests. Additionally, a novel collision-aware recursive regression module is introduced to update the network parameters recursively using the interpenetration distances calculated from the vertex-face and edge-edge tests.

Using a regression module, our model detects collisions. Finally, to provide a compact collision response, a novel self-supervised term is introduced. In summary, our main contributions are as follows: (1) propose a GNN with a collision-aware recursive regression module to effectively sense and respond to tool-object collisions; (2) a novel self-supervised collision term is introduced to reduce the interpenetration errors in unseen (that is, test) sequences and provide a more compact collision response; and (3) the proposed method was extensively evaluated in several common interactive simulation scenes with vertex-face, edge-face, and face-face collisions.

### Related works

This section reviews three main areas: deep simulation, CCD, and GNNs.

#### Deep simulation

Neural networks can be used as effective function approximators in physical systems. For linear elastic deformation, Luo et al. [3] proposed a highly reusable and efficient neural network-based nonlinear deformable simulation framework, which partially restores the force-displacement relationship by warping the simulated nodal displacement, and used a simplistic constitutive model to infer the linear elasticity. For nonlinear elastic deformation, Holden et al. [1] combined subspace simulation techniques with machine learning to support interactions with external objects. Romero et al. [4] used a model formula with nonlinear corrections applied to the local undeformable setting and decoupled internal and external contact-driven corrections.

The collision process is one of the physical simulation difficulties associated with deep learning techniques. The most basic method is to learn the implicit collision relations between collision objects. Teng et al. [5] managed self-collisions by applying forces on a sparse set of de-projected simulation points. They supported external collisions by allowing partial, albeit costly, full-space simulations in collision-prone mesh areas. Additionally, Tan et al. [6] presented a learning-based method that synthesizes collision-free 3D human poses. They decomposed whole-body collisions into groups of collisions between localized body parts using a bi-level autoencoder. Pfaff et al. [7] took advantage of the excellent explanatory capability of GNNs for graph datasets (mesh-based datasets), and their model can learn the dynamics of a wide range of physical systems, from cloth simulation over structural mechanics to fluid dynamics directly. In this study, a GNN is used as a base model because it can learn complete vertex-edge-face information.

#### CCD

CCD is widely applied in many areas, including physical-based simulation, computer-aided design/computer-aided manufacturings, and robot motion planning. Its main purpose is to use some form of the interpolating trajectory to check for collisions between two discrete positions of objects or primitives. A common method of CCD is to simply enclose the bounding volumes (BVs) at the beginning and end of a motion step using a swept volume. Axis-aligned bounding boxes are usually chosen for this method. Coming and Staadt [8] proposed a velocity-aligned discrete oriented polytopes as a type of swept volume for underlying spheres as BVs. Additionally, Redon et al. [9] proposed an oriented bounding boxes algorithm. Penetration depth-based detection is another method of CCD. The minimum distance is not a good measure for defining repelling forces, and computing the exact impact time using CCD is too time-consuming for real-time applications. Redon and Lin [10] estimated the local penetration depth on the graphics processing unit using the local penetration direction computed for these regions. Tang et al. [11] traced the contact features along their deforming trajectories and accumulated penalty forces along the penetration time intervals between overlapping feature pairs.

Choi et al. [12] presented a framework for the CCD of composite quadric models with piecewise linear or quadric surface patches as boundary surfaces and conic curves or line segments as boundary curves. Although these methods can effectively provide CDR in traditional physical simulations, deep simulations remain an open problem.

#### GNNs

GNNs have been shown to be effective representations for learning large-scale tasks [13]. A GNN can effectively learn knowledge representation [14], message passing [15], and encode long-range dependencies (video processing). GNNs also perform well in dynamic physical systems, such as for climate prediction [16], with an emphasis on individual objects [17] and their relations [18], partially observable systems [19], prevalent interactions within physical systems [14], hierarchically organized particle systems [20], or more generally physical simulation [7, 21, 22].

## Methods

**Fig. 1:**
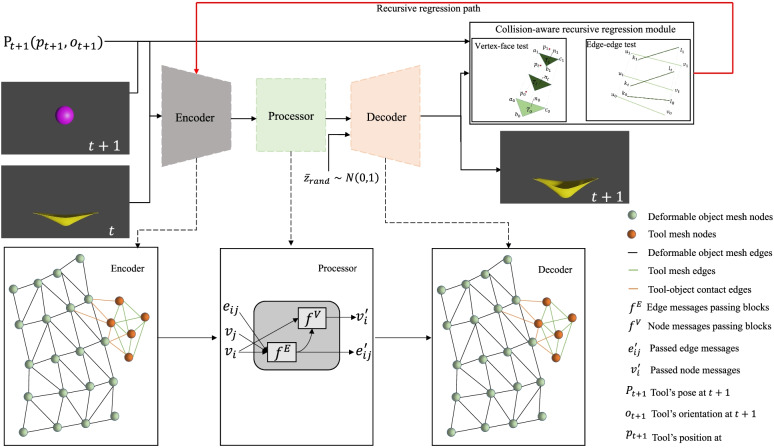
Overview of the proposed method. GNN is the base model. A collision-aware recursive regression module updates the network parameters recursively using interpenetration distances calculated from vertex-face and edge-edge tests. A novel self-supervised collision term (random latent space vector ${\overline{z}}_{rand}\sim$ (0, 1)) provides a more compact collision response

The objective of the study is to design a deep interactive physical simulation framework that can effectively address tool-object collisions. A GNN-based encoder-processor-decoder architecture was chosen as the baseline, which can provide complete vertex-edge-face information. To detect tool-object collisions, a collision-aware recursive regression module that uses interpenetration distances calculated from vertex-face and edge-edge tests to recursively update network parameters is introduced. Furthermore, a novel self-supervised collision term to provide a more compact collision response is introduced to reduce the interpenetration errors in unseen (that is, test) sequences. Figure 1 shows an overview of the proposed method.

### GNN-based architecture

**Fig. 2:**
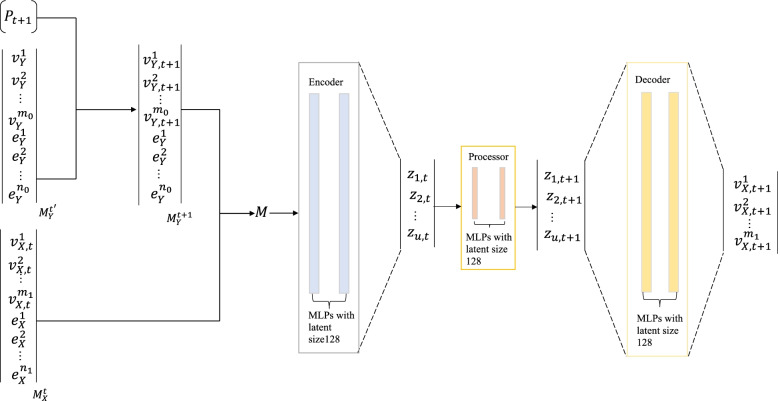
The proposed network configuration. *m*_0_, *m*_1_, *n*_0_, and *n*_1_ are the numbers of nodes and edges of the deformable object and tool. The encoder, processor and decoder have two latent layers with a latent size 128

A GNN-based encoder-process-decoder architecture is used to learn the dynamic information. GNNs, compared with other networks, can provide complete vertex-edge-face information. In particular, the dynamic information is encoded to a mesh graph, passed the messages on the mesh graph, and adapted the mesh discretization during the forward simulation. The mesh discretization information of the latent space contains the dynamic information of the system, and the mesh discretization information can be decoded to learn the dynamic information of the system. Figure 2 shows the network-specific configuration.

A combined mesh $M=\left({M}_X^t,{M}_Y^{t+1}\right)$ is the model input, where ${M}_X^t=\left({V}_X,{E}_X\right)$is the simulation mesh of the deformable object at time *t*, nodes *V*_*X*_ connected by mesh edges *E*_*X*_ an ${M}_Y^{t+1}\left({M}_Y^{t^{\prime }},{P}_{t+1}\right)=\left({V}_Y,{E}_Y\right)$ is the simulation mesh of tool at time *t* + 1, nodes *V*_*Y*_ connected by mesh edges *E*_*Y*_ . Additionally, *P*_*t* + 1_(*p*_*t* + 1_, *o*_*t* + 1_) is the pose of the tool (position *p*_*t* + 1_ and orientation *o*_*t* + 1_) at time *t* + 1 and ${M}_Y^{t^{\prime }}$ is the base tool mesh at time *t*^′^. We used *P*_*t* + 1_ and ${M}_Y^{t^{\prime }}$ to calculate the tool mesh ${M}_Y^{t+1}$ at time *t* + 1. Each node *i* ∈ *M* is associated with coordinates *u*_*i*_, additional dynamic information *q*_*i*_.

**Encoder:** The combined mesh is encoded into a multigraph *G* = (*V*, *E*). The nodes in the mesh correspond to the nodes in the graph, and the edges in the mesh correspond to the *E* in the graph. They are used to calculate the dynamic information inside the system. *E* handles dynamic information external to the system, such as collisions and contacts, which are the overall information of the system. An edge feature is defined as follows: ${e}_{ij}\left({e}_{ij}^W,{e}_{ij}^M\right)$, where ${e}_{ij}^M\in E$ and ${e}_{ij}^W={u}_i-{u}_j$, if |*u*_*i*_ − *u*_*j*_| < *r*_*W*_, *r*_*W*_ is the collision radius. If the world distance between two nodes is less than *r*_*W*_, the two nodes may collide. The node feature *v*_*i*_ is represented as a dynamic feature *a*_*i*_ and a one-hot vector of node types. Finally, the node and edge features are encoded by two latent layers multilayer perceptrons (MLPs) ϵ^*E*^, ϵ^*V*^ into a 128 dimensional hidden vector.

**Processor:** Message-passing blocks were used to pass messages on the mesh graph and adapt the mesh discretization during forward simulation. The processor consists of *L* identical message passing blocks, which generalizes GraphNet blocks to multiple edge sets, and *L* = 2 by default. Each block contains a separate set of network parameters and is sequentially applied to the output of the previous block, updating the edge *e*_*ij*_, and node *v*_*i*_ embeddings to ${e}_{ij}^{\prime }$ and ${v}_i^{\prime }$, respectively, by the following:

1 $${e}_{ij}^{\prime}\leftarrow {f}^E\left({e}_{ij},{v}_i,{v}_j\right),{v}_i^{\prime}\leftarrow {f}^V\left({v}_i,\sum_{j}{e}_{ij}^{\prime}\right)$$

where *f *^*E*^ and *f *^*V*^ are implemented using two latent-layer MLPs with residual connections. Then, the proposed model learns the dynamic information latent space at time *t* + 1, the key to which is to decode the latent dynamic information to the real physical space.

**Decoder:** To transform the latent dynamic information space into real physical dynamic information, a two latent-layer MLP, *δ*^*V*^, was used as a decoder to update the dynamic information of the nodes in the mesh by converting the latent node feature *v*^*i*^ at time *t* to the dynamic feature *a*^*i*^ of a deformable object at time *t*. The dynamic feature *a*^*i*^ is the derivative of the dynamic information *q*^*i*^ at time *t*. The forward Euler integration can be used to calculate the dynamic information ${q}_{t+1}^i$ at time *t* + 1. For first-order systems, ${q}_{t+1}^i={a}^i+{q}_t^i$, whereas for second-order systems, ${q}_{t+1}^i={a}_i+2{q}_t^i-{q}_{t-1}^i$.

Furthermore, to train a collision-aware model that learns dynamic information and tool-object collisions, the proposed GNN-based model loss is defined as follows:

2 $${L}_{GNN}={L}_q+{L}_{ccd}+{L}_{compact}$$

where *L*_*q*_ is the dynamic information loss defined as follows:

3 $${L}_q={\left|q-\overline{q}\right|}_2$$

*L*_*ccd*_ is the collision-aware recursive regression module’s continuous collision-detection loss, which is explained in detail in collision-aware recursive regression module section. *L*_*compact*_ is the self-supervised term loss that provides a compact collision response, which is explained in detail in self-supervised term section.

### Collision-aware recursive regression module

The message-passing architecture learns dynamic information. However, it is difficult to detect the collision information in the system. To address this problem, the architecture outputs are used as the inputs to the collision-aware recursive regression module to calculate the interpenetration distance and update the network parameters recursively. Additionally, a novel self-supervised collision term is introduced to provide a more compact collision response.

To calculate the interpenetration distance, a non-penetration continuous collision-detection filter that filters vertex-face collision and edge-edge collision pairs is used. The interpenetration distance is defined as the continuous collision-detection loss of the module as follows:

4 $${L}_{ccd}={\upxi}_{VF}+{\upxi}_{EE}$$

where ξ_*VF*_ and ξ_*EE*_ are the distances between vertex-face and edge-edge collision pairs, respectively.

**Fig. 3:**
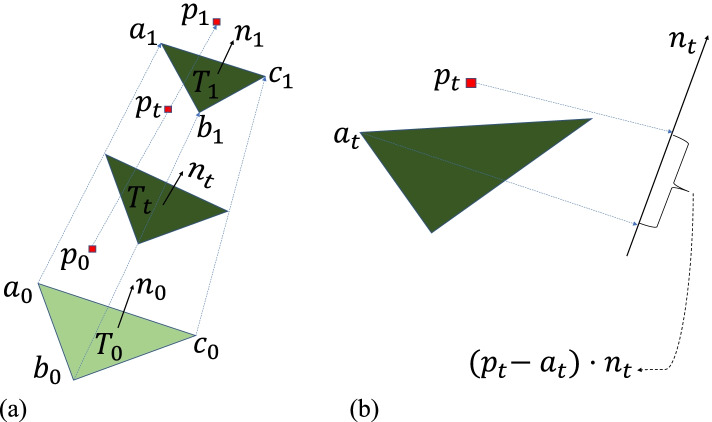
Vertex-face test: To perform a vertex-face test between a deforming triangle (defined by *a*_0_, *b*_0_, and *c*_0_ at *t* = 0, and *a*_1_, *b*_1_, and *c*_1_ at *t* = 1) and a moving vertex (defined by *p*_0_ at *t* = 0 and *p*_1_ at *t* = 1), coplanarity between the vertex and the triangle by finding a *t* (*t ∈* [0*,* 1]) when the projected distance along the normal vector of the triangle is equal to zero, that is, (*p*_*t*_ *− a*_*t*_) *· n*_*t*_ = 0 is checked. **a**: Deforming triangle *T* and deforming vertex *p*; **b**: Projected distance between *p*_*t*_ and *T*_*t*_

**Fig. 4:**
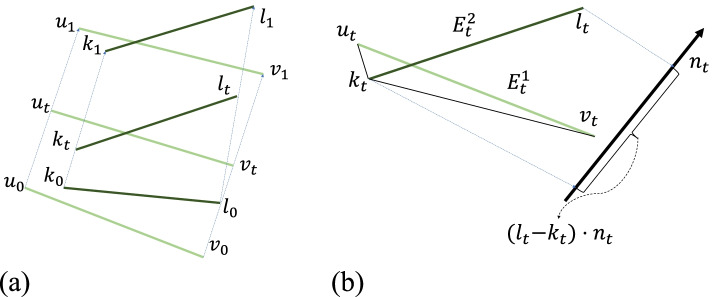
Edge-edge test: To perform an edge-edge test between the two edges *E*^1^ and *E*^2^ (defined by *u*_0_, *v*_0_, and *k*_0_, *l*_0_ at *t* = 0, and *u*_1_, *v*_1_, and *k*_1_, *l*_1_ at *t* = 1), the coplanarity conditions of these vertices by finding a *t* (*t ∈* [0*,* 1]) when the projected distance between *l*_*t*_ and the triangle defined by *k*_*t*_, *u*_*t*_ and *v*_*t*_ is equal to zero, that is, (*l*_*t*_ *− k*_*t*_) *· n*_*t*_ = 0 is checked

Traditional iterative continuous collision-detection algorithms are difficult to integrate into networks; therefore, a fast non-penetration continuous collision-detection filter [23] is chosen as the collision-aware recursive regression module to calculate the interpenetration distance. Furthermore, because of the high computational cost, a culling strategy based on the signed distance field (SDF) values is provided. The collision-detection module contains two terms: the vertex-face and edge-edge tests. Figures 3(a) and 4(a) show the vertex-face and edge-edge tests, respectively.

**Vertex-face test:** For a triangle *T*_*t*_ and a vertex *P*_*t*_ defined by the start and end positions during the interval [0, 1], these positions are linearly interpolated in the interval with respect to the time variable *t*. If the following four scalar values, *A*, *B*, $\frac{2\ast C+F}{3}$, and $\frac{2\ast D+E}{3}$ have the same sign, *T*_*t*_ and *P*_*t*_ will not be coplanar during the interval:

$${\displaystyle \begin{array}{c}A=\kern0.5em \left({p}_0-{a}_0\right)\kern0.5em {n}_0\\ {}B=\kern0.5em \left({p}_1-{a}_1\right)\kern0.5em {n}_1\\ {}C=\kern0.5em \left({p}_0-{a}_0\right)\kern0.5em \overline{n}\\ {}D=\kern0.5em \left({p}_1-{a}_1\right)\kern0.5em \overline{n}\\ {}E=\kern0.5em \left({p}_0-{a}_0\right)\kern0.5em {n}_1\\ {}F=\kern0.5em \left({p}_1-{a}_1\right)\kern0.5em {n}_0\end{array}}$$

where *n*_0_ is the normal of △*a*_0_*b*_0_*c*_0_, *n*_1_ is the normal of △*a*_1_*b*_1_*c*_1_, and $\overline{n}=\frac{n_0+{n}_1-\left(\overrightarrow{v_b}-\overrightarrow{v_a}\right)\times \left(\overrightarrow{v_c}-\overrightarrow{v_a}\right)}{2},\overrightarrow{v_a}$ is the vector of *a*_0_*a*_1_, $\overrightarrow{v_b}$ is the vector of *b*_0_*b*_1_, $\overrightarrow{v_c}$ is the vector of *c*_0_*c*_1_.

**Edge-edge test:** For two edges *E*^1^ and *E*^2^ defined by the start and positions during the interval [0, 1], these positions are linearly interpolated in the interval with respect to the time variable *t*. If the following four scalar values: *A*^′^, *B*^′^, $\frac{2\ast {C}^{\prime }+{F}^{\prime }}{3}$, and $\frac{2\ast {D}^{\prime }+{E}^{\prime }}{3}$ have the same sign, *E*^1^ and *E*^2^ will not be coplanar during the interval.

$${\displaystyle \begin{array}{c}{A}^{\hbox{'}}=\kern0.5em \left({l}_0-{k}_0\right)\kern0.5em {n}_0^{\hbox{'}}\\ {}{B}^{\hbox{'}}=\kern0.5em \left({l}_1-{k}_1\right)\kern0.5em {n}_1^{\hbox{'}}\\ {}{C}^{\hbox{'}}=\kern0.5em \left({l}_0-{k}_0\right)\kern0.5em {\overline{n}}^{\hbox{'}}\\ {}{D}^{\hbox{'}}=\kern0.5em \left({l}_1-{k}_1\right)\kern0.5em {\overline{n}}^{\hbox{'}}\\ {}{E}^{\hbox{'}}=\kern0.5em \left({l}_0-{k}_0\right)\kern0.5em {n}_1^{\hbox{'}}\\ {}{F}^{\hbox{'}}=\kern0.5em \left({l}_1-{k}_1\right)\kern0.5em {n}_0^{\hbox{'}}\end{array}}$$

where ${n}_0^{\prime }$ is the normal of △*u*_0_*k*_0_*v*_0_, ${n}_1^{\prime }$ is the normal of △*u*_1_*k*_1_*v*_1_, and ${\overline{n}}^{\prime }=\frac{n_0^{\prime }+{n}_1^{\prime }-\left(\overrightarrow{v_u}-\overrightarrow{v_k}\right)\times \left(\overrightarrow{v_v}-\overrightarrow{v_k}\right)}{2}$, $\overrightarrow{v_k}$ is the vector of *k*_0_*k*_1_, $\overrightarrow{v_u}$ is the vector of *u*_0_*u*_1_, $\overrightarrow{v_v}$ is the vector of *v*_0_*v*_1_.

The computation cost of every vertex-face and edge-edge pair is very large; therefore, the vertexes which SDF values that are smaller than a certain value before filtering are culled. The filtered vertex-face and edge-edge pairs that did not collide and the rest were defined as the collision pairs.

For vertex-face collision pairs, ${\upgamma}^{t_0,{t}_0+1}$ are vertex-face pairs during the interval frame [*t*_0_, *t*_0_ + 1], and the vertex-face pairs collide at time *t*_0_ + 1. Figure 3(b) shows the vertex-face distance at time interval [0, 1]. To reduce the number of vertex-face collision pairs, the distance between vertex-face pairs is reduced. ${D}_{vf}^{t_0,{t}_0+1}$ is defined as the distance between vertex-face pairs during the interval frame interval [*t*_0_, *t*_0_ + 1]. Therefore, the vertex-face loss is defined as follows:

5 $${\upxi}_{VF}=\mathit{\max}\left({D}_{vf}\right)$$

where

6 $${D}_{vf}=\left[{D}_{vf}^{0,1},{D}_{vf}^{1,2},\dots, {D}_{vf}^{t,t+1}\right]$$

For edge-edge collision pairs, ${\upeta}^{t_0,{t}_0+1}$ are edge-edge pairs during the interval frame [*t*_0_, *t*_0_ + 1], and the edge-edge pairs collide at time *t*_0_ + 1. Figure 4(b) shows the edge distance at time interval [0, 1]. To reduce the number of edge-edge collision pairs, the distance between edge-edge pairs is reduced. ${D}_{ee}^{t_0,{t}_0+1}$ is defined as the distance between edge-edge pairs during the interval frame interval [*t*_0_, *t*_0_ + 1]. Therefore, the edge-edge loss is defined as follows:

7 $${\upxi}_{EE}=\mathit{\max}\left({D}_{ee}\right)$$

where

8 $${D}_{ee}=\left[{D}_{ee}^{0,1},{D}_{ee}^{1,2},\cdots, {D}_{ee}^{t,t+1}\right]$$

### Self-supervised term

Using the learned dynamic information defined in GNN-based architecture section and the tool-object collision detection module in collision-aware recursive regression module section, a collision-aware model to learn the dynamic information and tool-object collision can be trained. However, there were interpenetration errors in the unseen (that is, test) sequence. This challenge is addressed by learning a compact collision response that reliably solves tool-object interpretations. To provide a compact collision response, the following self-supervised collision term is proposed:

9 $$L_{compact}=\xi_{Random}+L_{KL}$$

and

10 $${\upxi}_{Random}=\mathit{\max}\left(\Delta - SDF\left(D\left({\overline{z}}_{rand}\right),{P}_{t+1}\right),0\right)$$

where ${\overline{z}}_{rand}\sim N\left(0,1\right)$, Δ is the collision-free constraint threshold, *SDF*() is the signed distance field of the tool, *D*() is the decoder of our model, and *P*_*t* + 1_ is the pose of the tool at time *t* + 1. The self-supervised term samples the latent space and checks collisions against a constraint tool mesh using a self-supervised strategy (that is*,* ground truth positions are not needed for this term). This key ingredient allows for thorough sampling of the latent space and the learning of a compact collision response that reliably solves the tool-object interpenetration problem.

The self-supervised loss is derived from ref. [24], which requires a consistent distribution of the sampled latent space and training data. To enforce a normal distribution in the latent space, an additional term *L*_*KL*_ is included.

### Datasets

Generally, most mesh-based simulation methods are suitable for acquiring data for the proposed method. The inputs to the training procedure were a raw time series of frame-by-frame vertex positions and face indices. More details about the exact data acquisition process used in our results are provided.

**Fig. 5:**
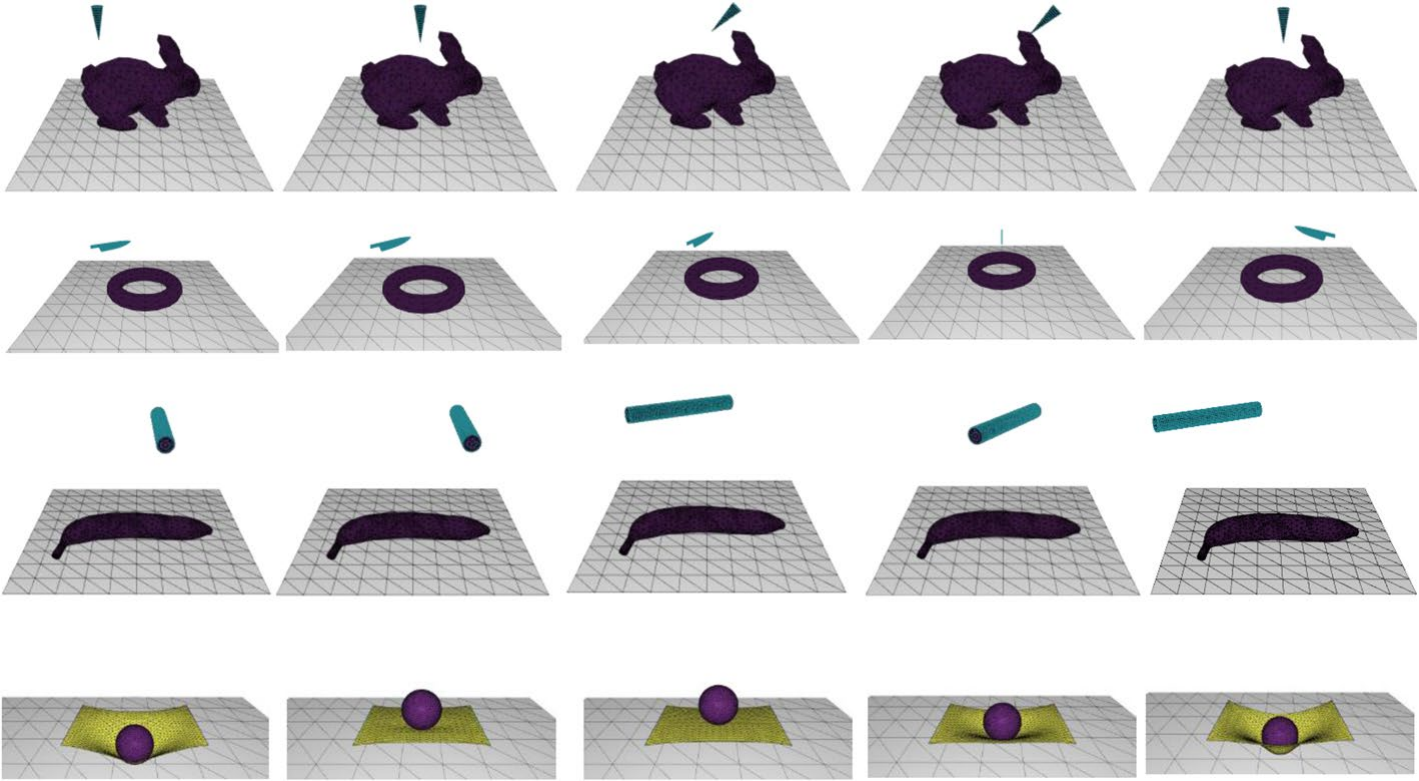
Applying IPC [25] to generate the datasets used in this study. The IPC library can output accurate CDR simulation results

All simulations were performed using the incremental potential contact (IPC) simulation library [25] and captured data at 25 fps. The datasets used are shown in Fig. 5. The IPC library can provide accurate CDR simulation results. The datasets used in this study involve vertex-face collisions (cone-bunny), edge-face collisions (knife-torus), and face-face collisions (sphere-mat and cylinder-banana). All datasets contain dynamic information (velocity), SDF values of the tools, vertex positions, and face information. The vertex-face collision datasets used in this study are cone-bunnies, which simulate a cone stabbing a bunny. The edge-face collision datasets used in this study are knife-torus, which simulate a knife cutting a rubber torus. The face-face collision datasets used in this study were a sphere-mat and cylinder-banana. The sphere-mat datasets simulated a rigid sphere falling onto a rubber mat, whereas the cylinder-banana datasets simulated a rigid cylinder pressing a banana. Table 1 shows the model complexity of the datasets.

**Table 1:** Execution time of the proposed method and IPC

Scene	Vertex	Edge	Face	IPC (ms)	The proposed method (ms)	Speedup
Cone-bunny	3022	9054	6036	2070	75	27.60
Knife-torus	5926	17,772	11,848	6380	60	127.60
Sphere-mat	4439	13,305	8870	2440	135	18.07
Cylinder-banana	3607	10,809	7206	1200	90	13.33

### Training

This section introduces the training software environment, normalization strategies, training noise, and optimization procedures used in this study.

**Software:** All models were implemented using TensorFlow1, Sonnet1, and the “Graph Nets” library.

**Normalization:** All input and target vectors elementwise were normalized to zero mean and unit variance, using statistics computed online during training. Normalization can lead to faster training and better performance. Preliminary experiments showed that normalization led to faster training, although the converged performance did not improve significantly.

**Training noise:** Modeling a complex and chaotic simulation system requires a model to mitigate error accumulation over long rollouts. Because the models in this study were trained on ground-truth one-step data, they were never presented with input data corrupted by this type of accumulated noise. This means that when a rollout is generated by feeding the model with its own noisy, previous predictions as input, the fact that its inputs are outside the training distribution may lead to more substantial errors and thus rapidly accumulate further error. A simple approach to make the model more robust to noisy inputs by corrupting the input positions of the model with Gaussian noise is used; thus, the training distribution is closer to the distribution generated during rollouts.

**Optimization procedures:** The model parameters were optimized over this loss with the Adam optimizer [26], using a nominal mini-batch size of one. A maximum of 1 × 10^5^ gradient update steps was performed with an exponential learning rate decay from 10^4^ to 10^6^. While models can be trained in fewer steps, this study avoided using aggressive learning rates to reduce variance across datasets and make comparisons across settings fairer.

## Results

This section demonstrates that our model can reliably process collisions in the physical system and conduct several experiments comparing the baseline, quantitative evaluation, and qualitative evaluation in different simulation scenes: face-face collisions, edge-face collisions, vertex-face collisions, and ablation studies. The reader is referred to the supplemental video for the corresponding animations. The proposed model runs on a PC with a central processing unit Intel E5–2637, 128 GB RAM, and a GTX 1080 Ti graphics card.

### Comparison

**Fig. 6:**
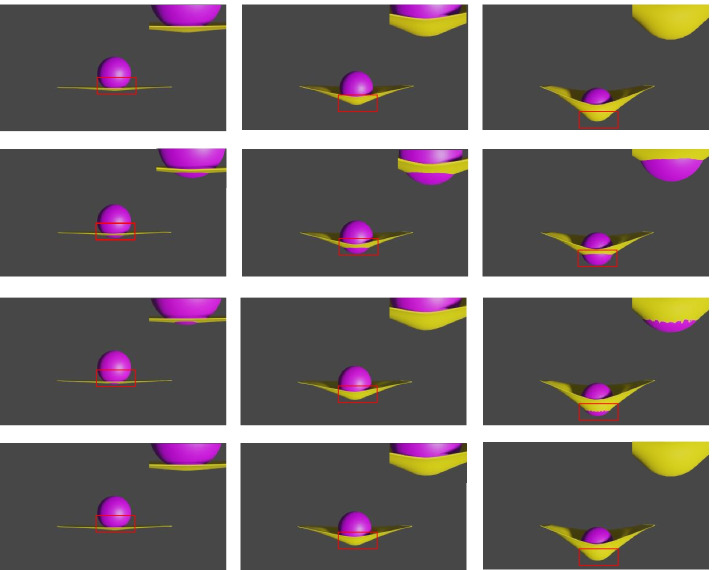
Comparison. The top row is the ground truth, the second row is the subphysics simulation result, the third row is the baseline simulation result, and the bottom row is the proposed method’s simulation result

A sphere-mat scene is chosen to demonstrate the advantage of the proposed method in processing collisions compared with the baseline. The proposed method is compared with subphysics [1] and a baseline. The baseline comes from meshgraphnets [7] without remeshing because remeshing changes the topology of the data, which is not conducive to evaluating collisions. Figure 6 shows a comparison between the proposed method and the baseline. The top row is the ground truth, the second row is the subphysics simulation result, the third row is the baseline simulation result, and the bottom row is the proposed method simulation result. The results show that their method has a large interpenetration area, whereas the proposed method has none. Clearly, the proposed method detects collisions in the physical system.

### Qualitative evaluation

**Fig. 7:**
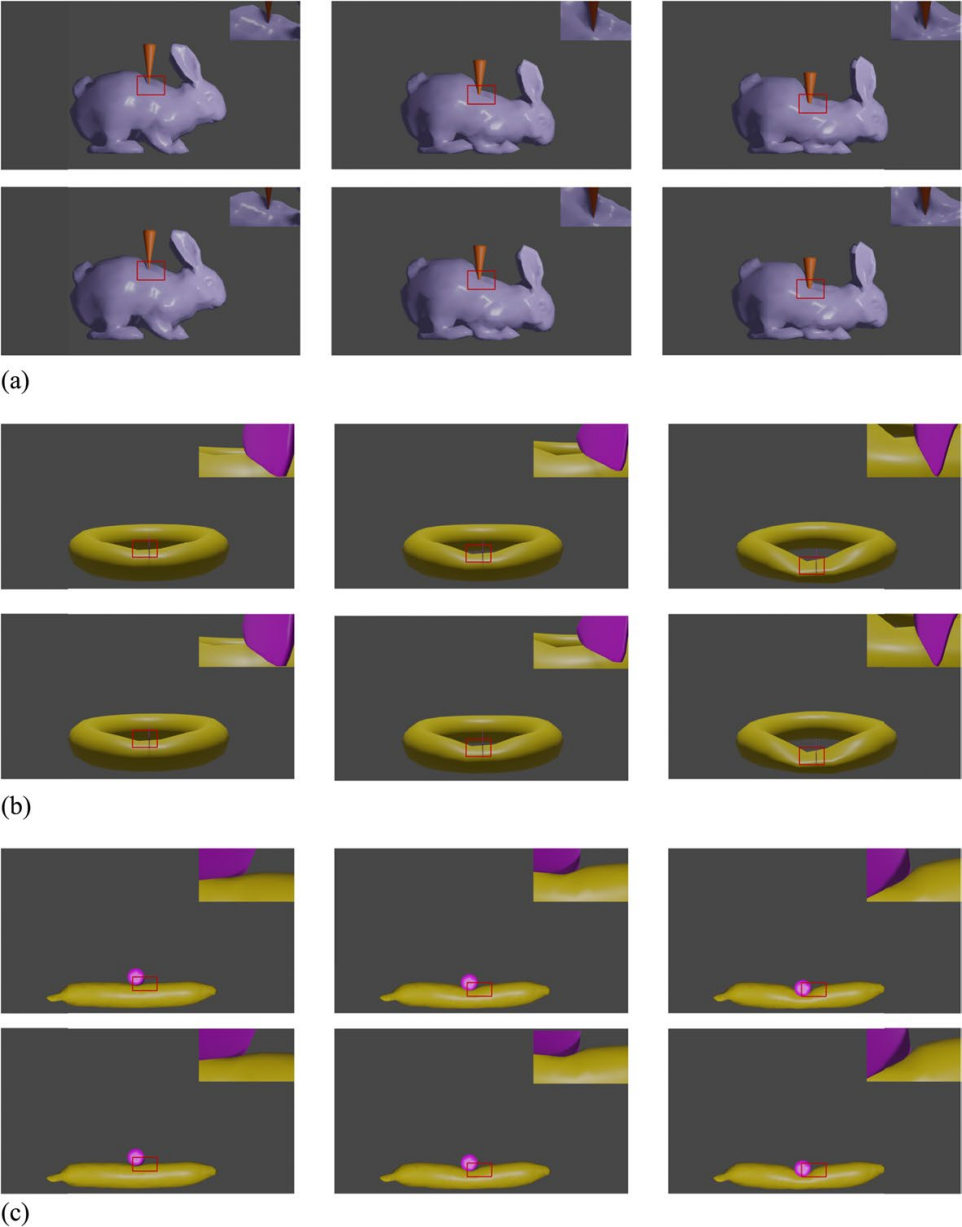
Qualitative evaluation. The proposed method is evaluated in three different collision scenes: vertex-face, edge-face, and face-face collisions. For each scene, the first row is the simulation result of the proposed method, and the second row is the ground truth. **a**: Vertex-face collisions; **b**: Edge-face collisions; **c**: Face-face collisions

To demonstrate the effectiveness of the proposed method in terms of quality, three different collision scenarios were defined: vertex-face collision, edge-face collision, and face-face collision. Figure 7(a) shows the vertex-face collision, Fig. 7(b) shows the edge-face collision, and Fig. 7(c) shows the face-face collision. In all three figures, the first row shows the simulation result of the proposed method, and the second row shows the ground truth. None of the three scenes had any interpenetration of the proposed method. Table 2 shows the quantitative evaluation of the collision elimination. The table shows that the proposed method can effectively eliminate collision errors. According to the results in the figures and table, the proposed method can process vertex-face, edge-face, and face-face collisions.

**Table 2:** Quantitative evaluation of collision elimination

Scene	*N*_*vf *_(%)	*d*_*vf*, *mean *_(%)	*d*_*vf*, *max *_(%)	*N*_*ee *_(%)	*d*_*ee*, *mean *_(%)	*d*_*ee*, *max *_(%)
Vertex-face collision	69.61	72.23	69.63	–	–	–
Edge-edge collision	62.50	53.67	49.84	–	–	–
Face-face collision	58.15	58.16	59.51	34.46	42.22	45.22

*N*_*vf*_, *d*_*vf*, *mean*_, *d*_*vf*, *max*_, *N*_*ee*_, *d*_*ee*, *mean*_ and *d*_*ee*, *max*_ represent mean vertex-vertex collision number, mean vertex-vertex collision interpenetration distance, max vertex-vertex collision interpenetration distance, mean edge-edge collision number, mean edge-edge collision interpenetration distance and max edge-edge collision interpenetration distance, respectively

### Quantitative evaluation

**Fig. 8:**
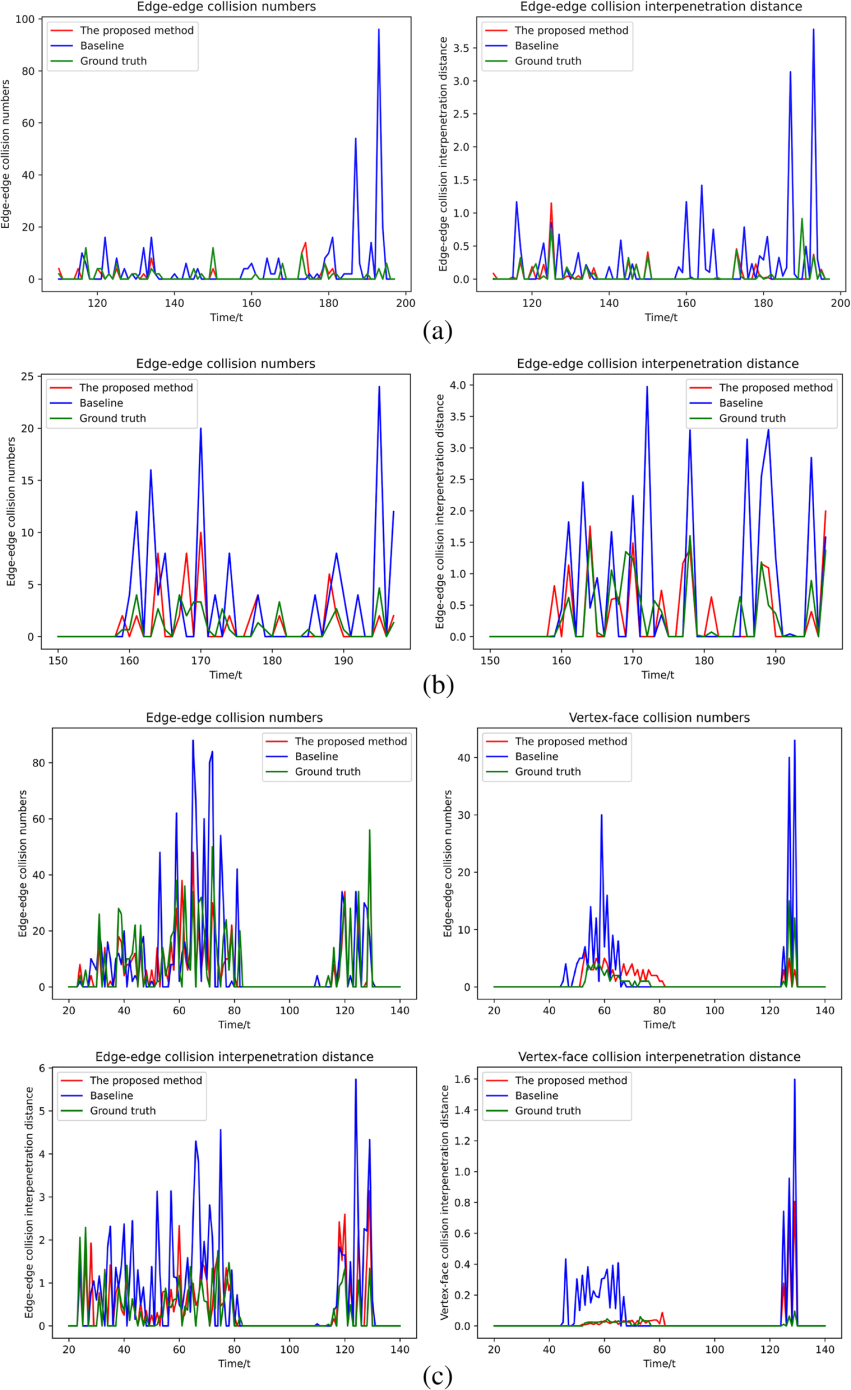
Quantitative evaluation. The proposed method is evaluated using the numbers and interpenetration distance of vertex-face and edge-edge collisions. **a**: Vertex-face collision evaluation; **b**: Edge-face collision evaluation; **c**: Face-face collision evaluation

To demonstrate the effectiveness of the proposed method, three different scenes of collisions were defined: vertex-face, edge-face, and face-face collisions. Four collision quantitative evaluations were used: vertex-face collision numbers, vertex-face collision interpenetration distance, edge-edge collision numbers, and edge-edge collision interpenetration distance to judge the effectiveness of the proposed method’s processing collision. Figure 8 shows four collision quantitative evaluation results for three collision scenes.

Because ref. [1] lacks a CCD module, it is excluded from the comparison. Clearly, for the four collision quantitative evaluations, the proposed method is fairly less accurate than the baseline [7] and is close to the ground truth. The results demonstrate that the proposed method can effectively reduce collision errors in physical systems.

### Ablation study

**Fig. 9:**
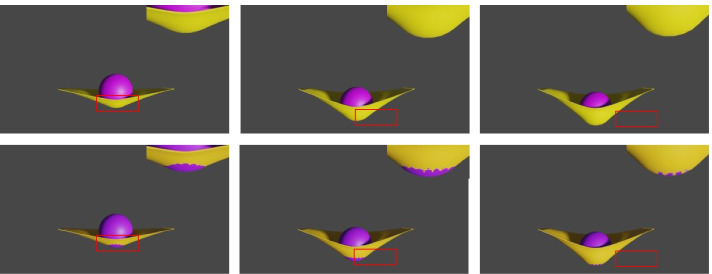
Ablation study. The first row is the proposed method’s simulation result, and the second row is the ablation simulation results

The self-supervised term was removed from the proposed method to demonstrate the effectiveness of the random latent space in completing CDR. Figure 9 shows the comparison results. The first row shows the proposed method’s simulation results, while the second row shows the ablation simulation results. The figure shows that there are interpenetrations if the self-supervised term is removed. The results show that using the self-supervised term to complete the collision response is crucial for the proposed method.

### Performance

The proposed method is compared with the ground truth physical simulator IPC to evaluate its performance. Table 1 presents the results of this comparison. As the table shows, the proposed method leverages IPC by at least one order of magnitude.

## Discussion

In this section, the advantages and challenges of the proposed method are discussed. The proposed method is compared with other state-of-the-art methods. Although the simulation results of the other methods have interpenetrations, the proposed method has none. Clearly, the proposed method detects collisions in the physical system better than the other methods. The proposed method was qualitatively and quantitatively evaluated in three collision scenarios: vertex-face, edge-face, and face-face collisions. The proposed method can visually produce no interpenetration results and effectively reduce the number of vertex-face collision and edge-edge collisions, resulting in visually excellent and physically accurate results. An ablation study was conducted to demonstrate the effectiveness of the random latent space for complete CDR. Some interpenetrations occur without a random latent space, demonstrating that our self-supervised term effectively reduces interpenetrations. Furthermore, compared to traditional CCD methods (IPC), the proposed method leverages at least one order of magnitude. In conclusion, to the best of our knowledge, the proposed deep learning-based framework can effectively address tool-object collisions and is a state-of-the-art method.

However, this study only focused on the interaction between a rigid tool and soft body. The penetration number of the vertex face and edge increases sharply as the model’s complexity increases. The existing framework does not support large-scale interactive simulation computations owing to the limitations of the existing storage and computational power of the workstation. Future studies should introduce multiscale representations to achieve large-scale interactive simulations.

## Conclusions

In this study, a deep interactive physical simulation framework that can effectively address tool-object collisions is presented. This was achieved using a GNN-based architecture and collision-aware recursive regression module to detect collisions. Additionally, a novel self-supervised collision term is introduced to provide a more compact collision response. The proposed method was extensively evaluated and the results demonstrated that it can effectively reduce interpenetration artifacts while ensuring high simulation efficiency. However, the trained model could only be applied to simulations using the same tool object. Further research must be conducted to enhance the generalizability of this study’s results. The existing framework does not support large-scale interactive simulation computations owing to the limitations of the existing storage and computational power of the workstation. Furthermore, future work must introduce multiscale representations to achieve large-scale interactive simulations.

## Supplementary Information

**Additional file 1.**

## Data Availability

The IPC library, available at https://github.com/ipc-sim/IPC, was used to generate simulation datasets.

## Abbreviations

GNN: Graph neural network
CDR: Collision detection and response
CCD: Continuous collision detection
BV: Bounding volume
MLP: Multilayer perceptron
SDF: Signed distance field
IPC: Incremental potential contact
